# Remarkable Divergence of the Sex-Linked Region between Two Wild Spinach Progenitors, *Spinacia turkestanica* and *Spinacia tetrandra*

**DOI:** 10.3390/biology11081138

**Published:** 2022-07-29

**Authors:** Hongbing She, Zhaosheng Xu, Helong Zhang, Jian Wu, Xiaowu Wang, Zhiyuan Liu, Wei Qian

**Affiliations:** Institute of Vegetables and Flowers, Chinese Academy of Agricultural Sciences, Beijing 100081, China; shehongbing_sp@163.com (H.S.); xuzhaosheng@caas.cn (Z.X.); zhanghelong@caas.cn (H.Z.); wujian@caas.cn (J.W.); wangxiaowu@caas.cn (X.W.)

**Keywords:** Y chromosome, sex-linked region (SLR), evolution, wild spinach progenitors

## Abstract

**Simple Summary:**

There remain substantial gaps in our understanding of the sex-linked region (SLR) in two wild spinach progenitors, *Spinacia turkestanica* and *S. tetrandra*, although SLR in cultivated spinach is well understood. Using 63 *Spinacia* accessions comprising 32 *S. oleracea*, 19 *S. turkestanica* and 12 *S. tetrandra*, we found that *S. oleracea* shared a similar SLR with *S. turkestanica*, while the SLR was remarkably divergent between *S. oleracea*/*S. turkestanica* and *S. tetrandra*. Additionally, the SLR increasingly expanded via accumulating repetitive sequences and was more conserved than the pseudoautosomal region (PAR) during the evolution of *S. tetrandra* to *S. oleracea*. The results obtained in this study provide a broader understanding of the evolution of SLR in *Spinacia* species.

**Abstract:**

The sex-linked region (SLR) plays an important role in determining the sex of a plant. The SLR of the Y chromosome, composed of a 14.1-Mb inversion and a 10-Mb Y-duplication region (YDR), was deciphered in *Spinacia oleracea* previously. However, our understanding of the SLR in its wild relatives, *S. turkestanica* and *S. tetrandra*, remains limited. In this study, we used 63 resequencing data from the three *Spinacia* species to infer the evolution of the SLR among the *Spinacia* species. In the SLR, all the cultivated spinach and *S. turkestanica* accessions were clustered into two distinct categories with both sexes, while the *S. tetrandra* accessions of both sexes were grouped. This suggests that *S. oleracea* shared a similar SLR with *S. turkestanica*, but not with *S. tetrandra*, which was further confirmed based on the population structure and principal component analysis. Furthermore, we identified 3910 fully sex-linked SNPs in *S. oleracea* and 92.82% of them were available in *S. turkestanica*, while none of the SNPs were adopted in *S. tetrandra*. Genome coverage in males and females supported the hypothesis that the YDR increasingly expanded during its evolution. Otherwise, we identified 13 sex-linked transposable element insertion polymorphisms within the inversion in both *S. oleracea* and *S. turkestanica*, demonstrating that the transposable element insertions might have occurred before the recombination suppression event of the inversion. The SLR was conserved compared with the pseudoautosomal region given that the genetic hitchhiking process occurred in the SLR during its evolution. Our findings will significantly advance our understanding of the characteristics and evolution of the SLR in *Spinacia* species.

## 1. Introduction

The origin and evolution of dioecy has been one of the most attractive topics for researchers [1]. Dioecy is rare in flowering plants, constituting approximately 6% of angiosperm species [2]. Sex chromosomes that determine the genders of individuals independently originated multiple times from autosomes [3]. Theoretical studies have predicted that recombination suppression is an indispensable event that occurs during the evolution of sex chromosomes, especially in the sex-linked region (SLR) [4,5]. The initial SLR might be small, but it increasingly expands by accumulating abundant repetitive sequences owing to non-recombination [6]. Recently, many SLRs have been identified [7,8,9,10,11], providing a broader understanding of the characteristics and the evolution of sex chromosomes.

Spinach (*Spinacia oleracea* L.) is an important vegetable crop in the Amaranthaceae family and has been cultivated for more than 2000 years [12]. Spinach is a dioecious species with an XY sex-determining system and is an ideal vegetable crop for investigating sex determination and sex chromosome evolution [13]. Quite a few sex-linked markers [13,14,15,16] and male-determining bacterial artificial chromosomes (BAC) [17] have been reported in spinach, which provides a basis for further identifying SLRs. Additionally, Yu et al. (2021) identified a non-recombining region with 39 bin markers co-segregating with sex, which are located at 45.2 cM of LG1, by constructing two high-density genetic maps. The region contains accumulated abundant repetitive sequences, and its X counterpart is estimated to be approximately 18.4 Mb [18]. Previously, we identified the X and Y haplotypes based on homozygous female (XX) and male (YY) genotypes in *S. oleracea*. Furthermore, we detected a 24.1-Mb SLR on the Y chromosome, corresponding to a 13-Mb inversion on the X chromosome, and both of them exhibited low recombination. Specifically, the 13-Mb inversion located on the X chromosome ranged from 85.8 to 98.8, corresponding to 86.5–95.8 Mb (termed IV2-1) and 105.9–110.6 Mb (termed IV2-2) on the Y chromosome. Therefore, the SLR on the Y chromosome consisted of a 14.1-Mb inversion and a 10-Mb Y-duplication region (YDR) that was a male-specific region, ranging 95.8–105.9 Mb, on the Y chromosome [19]. Recently, Cai et al. (2021) reported a chromosome-scale reference genome of monoecious spinach and identified an SLR on the X chromosome, which was consistent with our previous finding [20]. Until now, no sex-determining gene has been reported in spinach. A microRNA, sol-miR2550n, was recently identified and defined as a male-promoting factor [21].

There are two wild spinach species, *S. turkestanica* and *S. tetrandra*, which are defined as spinach ancestors and are expected to be a potential genetic resource for spinach breeding programs [22,23]. Previous investigations have demonstrated that *S. turkestanica* is more similar to the cultivated *S. oleracea* than *S. tetrandra*, based on nuclear and chloroplast genome levels [24,25]. Similarly to cultivated spinach, the two wild relatives are dioecious [22]. However, our understanding of the sex chromosomes and SLR in the wild relatives of spinach remains limited.

To explore the evolution of the SLR in *Spinacia* species, we analyzed 63 *Spinacia* whole-genome sequence data, comprising 32 *S. oleracea*, 19 *S. turkestanica*, and 12 *S. tetrandra*. By combining them with the haplotype-resolved Y chromosome in *S. oleracea*, we assessed the divergence level of the Y chromosome, structure, and evolution of the SLR between cultivated spinach and its two wild progenitors.

## 2. Results

### 2.1. Population Structure of S. oleracea, S. turkestanica, and S. tetrandra in the Sex-Linked Region

**Figure 1:**
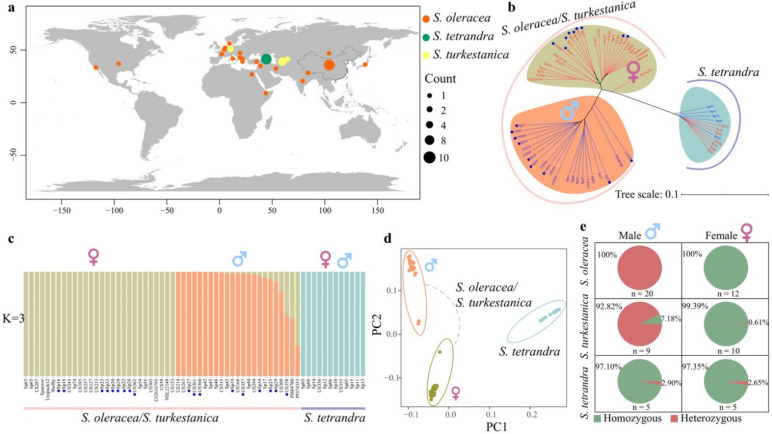
Population structure of the sex-linked region in *Spinacia*. (**a**) Geographic distribution of the 32 *Spinacia oleracea*, 19 *S. turkestanica*, and 12 *S. tetrandra* used in the study. (**b**) Phylogenetic tree based on 106,730 SNPs in the SLR on the Y chromosome. The accessions with red and blue colors represent females and males, respectively. The sample marked with a blue dot indicates *S. turkestanica*. (**c**) Population structure of the SLR in *Spinacia*. Each vertical bar represents an accession. The *S. turkestanica* accessions are marked with blue dots. (**d**) PCA of the SLR in the three *Spinacia*. (**e**) The distribution of 3910 fully sex-linked SNPs was identified using 32 *S. oleracea* in the two wild relatives.

To infer the evolution of the SLR among the three *Spinacia* species, we combined 29 publicly available data [20,26], and 34 newly sequenced data of *Spinacia* accessions, altogether making a set of 63 individuals, comprising 32 *S. oleracea* (12 males and 20 females), 19 *S. turkestanica* (10 males and 9 females), and 12 *S. tetrandra* (5 males and 7 females) (Table S1). The 63 representative *Spinacia* accessions came from 26 countries where all the sequenced spinach accessions existed (Figure 1a).

Based on the 63 *Spinacia* accessions, we identified 1,193,638 high-quality SNPs on the Y chromosome [19], of which 106,730 SNPs appeared in the SLR, ranging from 86.58 to 110.68 Mb, and the remaining SNPs existed in the pseudoautosomal region (PAR), which excludes the SLR on the Y chromosome. Based on these SNPs within the PAR, we constructed a neighbor-joining tree of *S. oleracea* and its two wild relatives, which was consistent with the previous finding that *S. turkestanica* shares a closer genetic relationship with *S. oleracea* (Figure S1) [24]. However, a phylogenetic tree of the three *Spinacia* species based on SNPs within the SLR showed that the *S. oleracea* and *S. turkestanica* accessions were clustered into two distinct categories with males and females, while all the females and males were grouped in *S. tetrandra* (Figure 1b). Our results strongly indicate that *S. oleracea* and *S. turkestanica* have similar SLRs. The population structure and principal component analysis (PCA) of the three *Spinacia* species in the SLR support this perspective (Figure 1c,d and Figure S2). Furthermore, we identified 3910 fully sex-linked SNPs that were heterozygous genotypes in all the males and homozygous genotypes in all the females in *S. oleracea* (Table S2). *S. oleracea* shared a substantial proportion (≥92.82%) of the fully sex-linked SNPs with *S. turkestanica*, while few SNPs *S. tetrandra* (Figure 1e and Figure S3) strongly reflected the differences in the SLRs between *S. tetrandra* and *S. oleracea*/*S. turkestanica*.

### 2.2. Evolution of the Y-Duplication Region among the S. oleracea and Two Wild Progenitors

**Figure 2:**
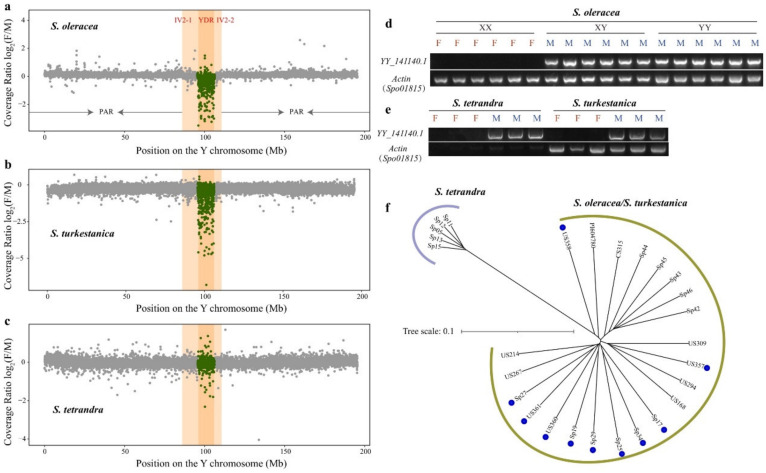
Characteristics of the Y-duplication region in cultivated spinach and two wild progenitors. (**a**) The gray dots show the sex-mapped read coverage ratio Log_2_(F/M) for 20 females and 12 males of *S. oleracea*. (**b**) The gray dots show the sex-mapped read coverage ratio Log_2_(F/M) for nine females and ten males of *S. turkestanica*. (**c**) The gray dots show the sex-mapped read coverage ratio Log_2_(F/M) for five females and five males of *S. tetrandra*. The read coverage per 20 kb bin was counted. YDR: Y-duplication region; IV: inversion; PAR: pseudoautosomal region. Complete male-specific conservation of *YY_141140.1* within the YDR in the genomes of (**d**) *S. oleracea* and (**e**) its two wild relatives, *S. turkestanica* and *S. tetrandra*. F: female; M: male. *Spo01815* is an actin gene in spinach. (**f**) Phylogenetic tree of 12 males of *S. oleracea*, 10 males of *S. turkestanica*, and 5 males of *S. tetrandra* based on the SNPs within the Y-duplication region. The accessions with blue dots are *S. turkestanica*.

YDR, a male-specific region on the Y chromosome, has been reported in many dioecious plants, and it increasingly expands due to recombination suppression [4,6]. Recently, we identified a 10-Mb YDR in *S. oleracea* [19]. The read coverage ratios of the females and males were determined to evaluate the divergence of the YDR among *S. oleracea*, *S. turkestanica*, and *S. tetrandra*. Our results showed that both *S. oleracea* and *S. turkestanica* exhibited the YDR (Figure 2a,b). In contrast, the read coverage ratios of the females and males did not show significant differences between the PAR/IV2 and YDR in *S. tetrandra*, suggesting a smaller or absent YDR in *S. tetrandra* than those in *S. oleracea* and *S. turkestanica* (Figure 2c). Thus, to deeply survey whether *S. tetrandra* accessions have YDR, we amplified the complete genome sequence of *YY_141140.1*, located at YDR of *S. oleracea* [19], in both sexes of the *S. oleracea*, *S. turkestanica*, and *S. tetrandra* accessions. The result showed that the presence of *YY_1141140.1* is restricted to males in all *Spinacia* species tested, further demonstrating that *S. tetrandra* harbored a smaller male-specific region (Figure 2d,e and Figures S8 and S9 and Table S3) and it might have expanded stepwise during the evolution of *S. tetrandra* to *S. turkestanica*, and *S. oleracea*.

A total of 16,366 high-quality SNPs were identified within the YDR-based 12 males of *S. oleracea*, 10 males of *S. turkestanica*, and 5 males of *S. tetrandra*. A neighbor-joining tree of these males in the YDR showed a remarkable divergence between *S. tetrandra* and *S. turkestanica*/*S. oleracea*. In contrast, both *S. oleracea* and *S. turkestanica* clustered, suggesting a recent divergence (Figure 2f). YDR is referred to as a vital region because many sex-determining genes are found within the region [27,28,29]. Among the 16,366 SNPs within the YDR, only 1.53% (251) were exonic SNPs and 2.84% (465) were intronic SNPs because of the YDR with abundant repetitive sequences (92.32%) and low gene-density (49 genes) (Figure S4) [19]. Twenty-eight out of 49 genes exhibited exonic SNPs, comprising 86 synonymous, 155 nonsynonymous, and 10 stopgains, and 30 genes bore intronic SNPs. Altogether, 37 out of 49 genes exhibited SNPs, suggesting that the remaining 12 genes might be more conservative (Tables S4 and S5 and Figure S5).

### 2.3. Landscapes of Transposable Element Insertion Polymorphisms in S. oleracea Sex Chromosomes

**Figure 3:**
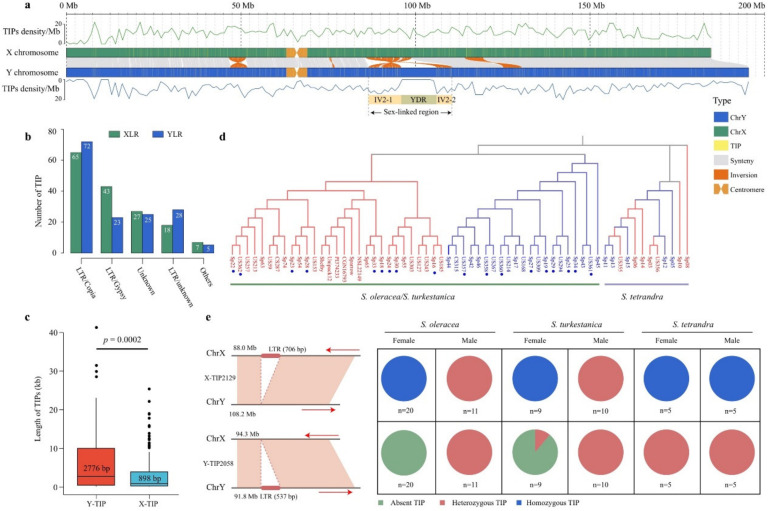
Identification of transposable element insertion polymorphisms (TIPs) in the sex-linked region. (**a**) Distribution of TIPs on the sex chromosomes in spinach. The top and bottom lines indicate TIP densities per Mb on the X and Y chromosomes, respectively. YDR: Y-duplication region; IV: inversion region. (**b**) Number of TIPs per TE family in the sex-linked region. XLR: X-linked region; YLR: Y-linked region. (**c**) Boxplot of the length of the TIPs detected in the sex-linked region. Y-TIP represents TIP on the Y-linked region. X-TIP represents TIP on the X-linked region. Significant difference analysis was performed using Student’s *t*-test. (**d**) Phylogenetic tree of 62 *Spinacia* accessions based on TIPs in the SLR. The accessions with red and blue colors represent females and males, respectively. The accession marked with a blue dot indicates *S. turkestanica*. (**e**) An example of TIP on the XLR and YLR in 62 *Spinacia* accessions. The right arrow represents the plus strand, while the left arrow represents the minus strand.

To deeply survey the landscape of transposable element (TE) insertion polymorphisms (TIPs) in spinach sex chromosomes, where the SLR is more likely to accumulate TEs [4,30], we identified 3616 TIPs, comprising 2053 TE insertions on the X chromosome (termed X-TIP), and 1563 TE insertions on the Y chromosome (termed Y-TIP) (Figure 3a; Tables S6 and S7). A total of 1571 (43.44%) TIPs were long terminal repeats (LTR)/Copia, which is a major LTR type in spinach [19,20] (Figure S6). For the SLR, we identified 313 TIPs, comprising 160 X-TIPs and 153 Y-TIPs, and 137 (43.76%) TIPs were LTR/Copia, which is consistent with the TIPs on the whole sex chromosomes (Figure 3b). Additionally, the SLR shared substantially higher TIP densities than the PAR (Student’s *t*-test, *p* = 0.002), while there was no significant difference between the X-TIP and Y-TIP densities in the SLR (Student’s *t*-test, *p* = 0.631). The Y-TIPs (median length = 2776 bp) appeared longer than the X-TIPs (median length = 898 bp) in the SLR (Student’s *t*-test, *p* = 0.0002) (Figure 3c).

The genotypes of 63 *Spinacia* accessions based on TIPs on the sex chromosomes was determined, as described by Cai et al. (2022) [31]. One sample (PI604780) was removed because of the high missing rate. At least 1776 TIPs on the sex chromosomes were genotyped using the 62 *Spinacia* accessions, and 180 out of the 1776 TIPs were located in the SLR. Similar to the phylogenic tree constructed using SNPs in the SLR, the phylogenic tree of the 62 *Spinacia* accessions based on TIPs in the SLR also showed that samples from *S. oleracea* and *S. turkestanica* were grouped into two distinct clades with females and males, whereas accessions from *S. tetrandra* were not (Figure 3d). Furthermore, we identified 13 fully sex-linked TIPs in *S. oleracea* and *S. turkestanica* (Figure S7). For example, X-TIP2129 and Y-TIP2058 shared a heterozygous genotype in all the males because the males are heterogametic (XY), and X-TIP2129 shared a homozygous genotype in the homogametic (XX) females, while Y-TIP2058 was absent in almost all females in *S. oleracea* and *S. turkestanica* (Figure 3e). However, the 13 sex-linked TIPs could not cosegregate with the sexes in *S. tetrandra*, suggesting recombination suppression in the SLR might have occurred after the 13 TE insertion, thus forming a conserved TIP in *S. oleracea* and *S. turkestanica*.

### 2.4. Patterns of the Y Chromosome Divergence between S. oleracea and Its Two Wild Progenitors

**Figure 4:**
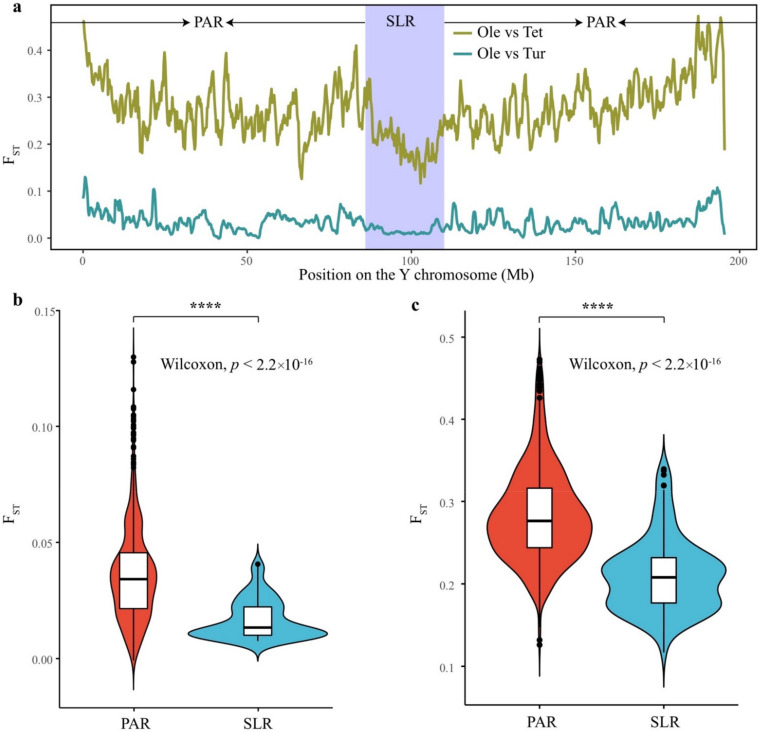
Pairwise *F*_ST_ between *S. oleracea* and its two wild progenitors on the Y chromosome. (**a**) The *F*_ST_ values between *S. oleracea* and its two wild relatives. Comparison of the *F*_ST_ values between the PAR and SLR in (**b**) *S. oleracea* vs. *S. turkestanica* and (**c**) *S. oleracea* vs. *S. tetrandra*. The Wilcoxon test was used for comparison between the two regions. PAR: pseudoautosomal region; SLR: sex-linked region. Ole: *S. oleracea*; Tet: *S. tetrandra*; Tur: *S. turkestanica*. The symbol **** indicates significant difference at the *p* < 2.2 × 10^−16^.

All the *S. oleracea* and *S. turkestanica* accessions clustered based on variants in the SLR, while they separately clustered in the PAR (Figure 1b and Figure S1). *S. tetrandra* did not cluster with *S. oleracea* and *S. turkestanica* in the PAR or SLR, suggesting a high genetic divergence between *S. tetrandra* and *S. oleracea*/*S. turkestanica*. To quantify the differences in the Y chromosome among the three *Spinacia* species, the population differentiation index (*F*_ST_) value between the *S. oleracea*, *S. turkestanica*, and *S. tetrandra* accessions were estimated. As shown in Figure 4a, *F*_ST_ for the Y chromosome was low between *S. oleracea* and *S. turkestanica* (mean *F*_ST_ = 0.033) and strikingly high for *S. tetrandra* (mean *F*_ST_ = 0.273) (Table S8).

The SLR exhibited a significant lower *F*_ST_ value than the PAR for both *S. oleracea* vs. *S. turkestanica* and *S. oleracea* vs. *S. tetrandra* (Figure 4b,c). This finding indicates that the SLR was a more conserved region than the PAR. The genetic hitchhiking process that occurs in a suppressed recombination region could result in low within-population diversity in the Y-linked region [32]. Previously, we confirmed that recombination in the SLR on both the X and Y chromosomes was suppressed [19]. Thus, the low *F*_ST_ values in the SLR (mean *F*_ST_ = 0.061 and 0.209 for *S. oleracea* vs. *S. turkestanica* and *S. tetrandra*, respectively) were likely to be a property of the Y chromosome.

## 3. Discussion

A comparison of cultivated spinach and its wild relatives is an available approach for studying the evolution of sex chromosomes and their dynamic structure [32]. In this study, we first inferred that *S. turkestanica* exhibited an SLR similar to that of *S. oleracea*, while *S. tetrandra* did not. Sharing almost fully sex-linked SNPs/TIPs, a size similar to that of the YDR, and a low *F*_ST_ value in the SLR between *S. oleracea* and *S. turkestanica* strongly suggests a very recent divergence of the SLR (Figure 1e, Figure 3e and Figure 4). However, a substantially diverged SLR was observed between *S. tetrandra* and *S. oleracea* due to a long enough evolutionary time [24,25]. Specifically, the fully sex-linked variants of the IV2 region for *S. oleracea*/*S. turkestanica* were not available in *S. tetrandra*, indicating that the IV2 region did not exist in *S. tetrandra*. Assembling the *S. tetrandra* genome will confirm this in the future.

Based on the plant chromosome evolution process, the sex-determining locus occurred first, followed by recombination suppression close to the sex-determining locus, resulting in a stepwise expanded SLR [5,6]. The wild relatives of the *S. tetrandra* possess smaller YDRs than those of *S. oleracea* and *S. turkestanica* (Figure 2c,d), and a male-specific gene *YY_141140.1* within the YDR was identified among the three *Spinacia* species (Figure 2d,e). Thus, the conserved male-specific gene within the YDR might be referred to as a potential sex-determining gene in spinach. As described in kiwifruit (*Actinidia chinensis*), the sex-determining genes, *SyGl* and *FrBy*, are specifically present in a wide variety of male *Actinidia* species [27,29].

Recombination suppression could facilitate plant sex chromosome evolution, prevent neuter individuals (both male and female sterility) from existing in the population and maintain the stable characteristics of dioecious plants [4,6,33]. In our understanding, *S. tetrandra* is dioecious and has no neuter individuals [22]; thus, the initial recombination close to the sex-determining locus might be suppressed in *S. tetrandra*. The SLR is expected to accumulate abundant repetitive sequences due to suppressed recombination [34]. Here, we also found significantly higher TIPs in the IV2 region than in the PAR (Figure 3a). Importantly, 13 fully sex-linked TIPs within the IV2 regions of *S. oleracea* and *S. turkestanica* were obtained (Figure 3e), suggesting that the conserved TIPs occurred before the recombination suppression event in the IV2 region. Furthermore, we speculated that the nonrecombination regions in the IV2 region originated from the 14.1-Mb large-scale inversion as chromosomal rearrangements (particularly inversion), which could result in recombination suppression, such as in papaya [35], humans [36], and ostrich [37]. Our findings show that excluding the 13 fully sex-linked TIPs, the remaining TE insertions on the sex chromosomes may have originated from the inversion.

During the evolution of *S. tetrandra* to cultivated spinach, the SLR was more conserved than the PAR (Figure 4). Furthermore, the phylogenetic relationship of the *Spinacia* species in the SLR did not correlate with the geographic regions (Figure 1a,b) although a small number of accessions were collected, further indicating a conserved SLR. The SLR, however, exhibited significant divergence between *S. tetrandra* and *S. oleracea*/*S. turkestanica*; thus, we hypothesized that there might be other *Spinacia* species between *S. tetrandra* and *S. turkestanica* that we did not collect. Additionally, given the limited sequencing technology in the study, we could not decipher the detailed evolution process of the SLR in the *Spinacia* species, including whether the TEs in *S. oleracea* increased compared with those in *S. turkestanica* and how the YDR was formed during evolution. High-quality genomes of the two wild progenitors will contribute to an extensive study of these in the future.

## 4. Conclusions

In the present study, we used 63 resequencing data from the three *Spinacia* species (cultivated and its two wild progenitors) to survey the evolution of the sex-linked region (SLR). Phylogenetic tree, population structure, and PCA analysis indicated that *S. oleracea* and *S. turkestanica* have similar SLR, while remarkably divergent SLR were found between *S. oleracea*/*S. turkestanica* and *S. tetrandra*. Additionally, *S. tetrandra* harbored a smaller male-specific region than that in *S. oleracea* and *S. turkestanica*. The SLR was conserved compared with the PAR during the evolution of sex chromosomes in *Spinacia* species. Further, we found 13 sex-linked TIPs that were inserted before the recombination suppression event on the sex chromosomes. Altogether, our results provide a basis to deeply investigate the evolution of sex chromosomes in the *Spinacia* species.

## 5. Materials and Methods

### 5.1. Plants Materials

A total of 34 individual *Spinacia species*, comprising 5 females and 5 males of *S. tetrandra*; 8 females and 6 males of *S. turkestanica*; and 5 females and 5 males of *S. oleracea*, were used for resequencing in this study. Among them, *S. tetrandra* and *S. turkestanica* accessions from the U.S Department of Agriculture (https://www.usda.gov; accessed on 23 May 2017) were introduced. The remaining *S. oleracea* were cultivated by the Spinach Breeding Group at the Institute of Vegetables and Flowers (IVF) of the Chinese Academy of Agricultural Sciences (CAAS). All the above plants were planted in the field at the IVF, CAAS, in spring 2018.

To collect as many spinach accessions as possible from different countries, 22 *Spinacia* accessions, comprising 15 *S. oleracea*, 5 *S. turkestanica*, and 2 *S. tetrandra* accessions, were downloaded from a previous investigation [20]. Meanwhile, seven *S. oleracea* accessions were also obtained [26]. A total of 63 *Spinacia* accessions from 26 countries were used in this study. The detailed information is summarized in Supplementary Table S1.

### 5.2. DNA Extraction and Whole-Genome Resequencing

Fresh leaves from each individual were collected and stored in liquid nitrogen. Genomic DNA was extracted using the DNeasy plant mini kit (Qiagen, Frankfurt, Germany). Paired-end (PE) Illumina libraries with an average insert size of 300 bp were constructed using the Illumina Genomic DNA Sample Preparation kit according to the manufacturer’s instructions (Illumina, San Diego, CA, USA). Then sequencing was performed using a HiSeq 2500 instrument (Illumina, San Diego, CA, USA) to generate 150-bp PE reads, representing an average of 10× coverage.

### 5.3. Read Mapping and Variant Calling

Raw PE reads from 63 *Spinacia* plants were processed to remove adapters and low-quality sequences using fastp (v0.20.0) with the parameter ‘-q 20’ [38]. Cleaned PE reads were aligned to the Sp_YY_v1 genome [19] using Burrows–Wheeler Aligner (v0.7.17) [39] with default parameters. Variants were identified using BCFtools (v1.8) [40] with the parameters ‘-q 20 -Q 30 -C 50’ and filtered using VCFtools (v0.1.16) [40] with the parameters ‘-maf 0.05, -mac 4 -minQ 30 -max-missing 0.9’. The annotation of the SNPs was performed using annovar [41]. Based on the variants of the 63 *Spinacia* accessions, the genders of the accessions collected from others were determined using a fully sex-linked marker, D4.3, used in spinach [16]. Reads coverage were calculated using BedTools (v2.26.0) with parameter settings ‘coveragell-bga’ [42].

### 5.4. Amplification of Gene within the YDR among the Three Spinacia Species

To determine whether *YY_141140.1* is male-specific in the Spinacia species, we amplified the genes in 18 S. oleracea (from inbred line 10S15 [19]), six *S. turkestanica* (Sp16, Sp18, Sp22, Sp25, Sp27, and Sp29), and six S. tetrandra accessions (Sp03, Sp06, Sp08, Sp11, Sp12, and Sp13). The primers of *YY_141140.1* and actin were designed using the online software Primer 3 (v0.4.0; https://bioinfo.ut.ee/primer3-0.4.0/; accessed on 3 March 2022). The polymerase chain reaction (PCR) was performed in a total reaction volume of 10 µL containing 5 µL of 2xTaq Master Mix (CoWin Biosciences, Taizhou, China), 0.25 µL of forward and reverse primers each, respectively, 3.6 µL of ddH_2_O and 1 µL of DNA/cDNA. The reaction was performed on a Veriti 96 Well Thermal Cycler (Applied Biosystems, Foster City, CA, USA) under the following conditions: 5 min at 94 °C, followed by 35 cycles of 30 s at 94 °C, 30 s at 60 °C, and 40 s at 72 °C. The result was assessed through electrophoresis with 1.5% agarose gels.

### 5.5. Population Analysis

A neighbor-joining phylogeny was constructed based on the *P* distance matrix calculated using VCF2Dis (v1.4.3) (https://github.com/BGI-shenzhen/VCF2Dis; accessed on 23 March 2022). PCA was performed using PLINK (v1.90b4.6; http://pngu.mgh.harvard.edu/purcell/plink/; accessed on 23 March 2022). The population differentiation indexes (*F*_ST_) across the Y chromosome were calculated in each 1000-kb window with a step size of 200 kb using VCFtools (v0.1.16) [40]. The population structure of the SLR was analyzed with the cluster number *K* ranging from 1 to 7 using ADMIXTURE (v 1.3.0) [43]. Each *K* was run 10 times, and the correct *K* value was chosen using ADMIXTURE’s cross-validation procedure.

### 5.6. Identification of Transposable Element Insertion Polymorphisms (TIPs)

To identify TIPs between the X and Y chromosomes in spinach, we first aligned the X chromosome to the Y chromosome from the Sp_XX_v1 and Sp_YY_v1 assemblies [19] using Mummer (v4.0.0rc1) with the parameter settings “-g 1000-c 90-l 40” [44]. The alignment block was then further filtered and one-to-one alignment was identified using delta-filter with the parameter setting “-1-i 90”. Then, the potential insertion and deletion (≥50 bp) relative to the Y chromosome were identified using show-diff in Mummer (v4.0.0rc1). Furthermore, the insertion and deletion sequences overlapped with the gap region in the respective sex chromosomes, and sequences with feature type “BRK” were ruled out. Meanwhile, the insertion and deletion in the syntenic regions were used in this study. Finally, the sequences of the insertion and deletion were mapped to the *S. oleracea* TE library [19] using BLASTN (v2.9.0). When the identification and coverage of the alignment was greater than 80%, then the insertion or deletion was defined as a TE insertion on the X (X-TIP) and Y (Y-TIP) chromosomes, respectively.

### 5.7. Genotyping of TIPs Using 63 Spinacia Accessions

We estimated the genotypes of each *Spinacia* accession as described by Cai et al. (2022) [31]. Specifically, we first defined each TE insertion sequence and their 1 kb upstream and downstream as the TE reference. Then, each clean PE read of the 63 *Spinacia* accessions was mapped to the TE reference using BWA-MEM (0.7.17-r1188) with parameter settings “-T 20-Y”. Next, based on the alignment position of the TE insertion, the genotype of each accession was obtained. Finally, the genotypes of TIPs with minor allele frequency (MAF) ≤0.05 and missing ≥0.08 were filtered.

### 5.8. Identification of Fully Sex-Linked SNPs/TIPs

For the SNPs on the Y chromosome, we defined one SNP as a fully sex-linked SNP when the genotype of the SNP was “0/1” in all males and “1/1” in all females. “0” indicated that the allele was consistent with the reference (the Y chromosome), while “1” indicated that the allele was consistent with the alternative. For the X-TIPs, one TIP was regarded as a fully sex-linked TIP when all the females appeared as homologous X-TIP, while all of the males appeared as heterozygous X-TIP. In contrast, one Y-TIP was regarded as a fully sex-linked TIP when the TIP was absent in all the females while sharing a heterozygous Y-TIP in all the males.

## Data Availability

The resequencing reads used in the study have been deposited in the Genome Warehouse in the BIG Data Center, Beijing Institute of Genomics, Chinese Academy of Sciences, under accession number CRA004067, and are publicly accessible at http://bigd.big.ac.cn (accessed on 4 April 2022).

## Supplementary Materials

The following supporting information can be downloaded at: https://www.mdpi.com/article/10.3390/biology11081138/s1, Figure S1: Phylogenetic tree of 63 *Spinacia* accessions based on SNPs within the pseudoautosomal region (PAR); Figure S2: The cross-validation (CV) error for different *K* values in admixture analysis; Figure S3: Fully sex-linked SNPs in *S. oleracea*, *S. turkestanica*, and *S. tetrandra*; Figure S4: Annotation of SNPs within the Y-duplication region; Figure S5: Venn diagram of genes with exonic or intronic SNPs in the Y-duplication region; Figure S6: Number of detected TIPs per TE family; Figure S7: Phylogenetic tree based on 13 conserved TIPs in 62 *Spinacia* accessions; Figures S8 and S9: Original images of full amplification of *YY_141140.1*; Table S1: List of 63 *Spinacia* accessions; Table S2: Fully sex-linked SNPs in *S. oleracea*; Table S3: Primer sequences used in this study; Table S4: Exonic SNPs within the Y-duplication region; Table S5: Intronic SNPs within the Y-duplication region; Table S6: TE insertion on the ChrY chromosome; Table S7: TE insertion on the X chromosome; Table S8: *F*_ST_ value between *S. oleracea* and the two wild progenitors on the Y chromosome.
