# Supplementary material for: Remarkable Divergence of the Sex-Linked Region between Two Wild Spinach Progenitors, Spinacia turkestanica and Spinacia tetrandra
Source: Biology (Basel). 2022 Jul 29;11(8):1138. doi: 10.3390/biology11081138 (PMC9404990; doi:10.3390/biology11081138)
Supplement: Supplementary file 1 [file biology-11-01138-s001.zip › Supplementary Figure S9.pdf]

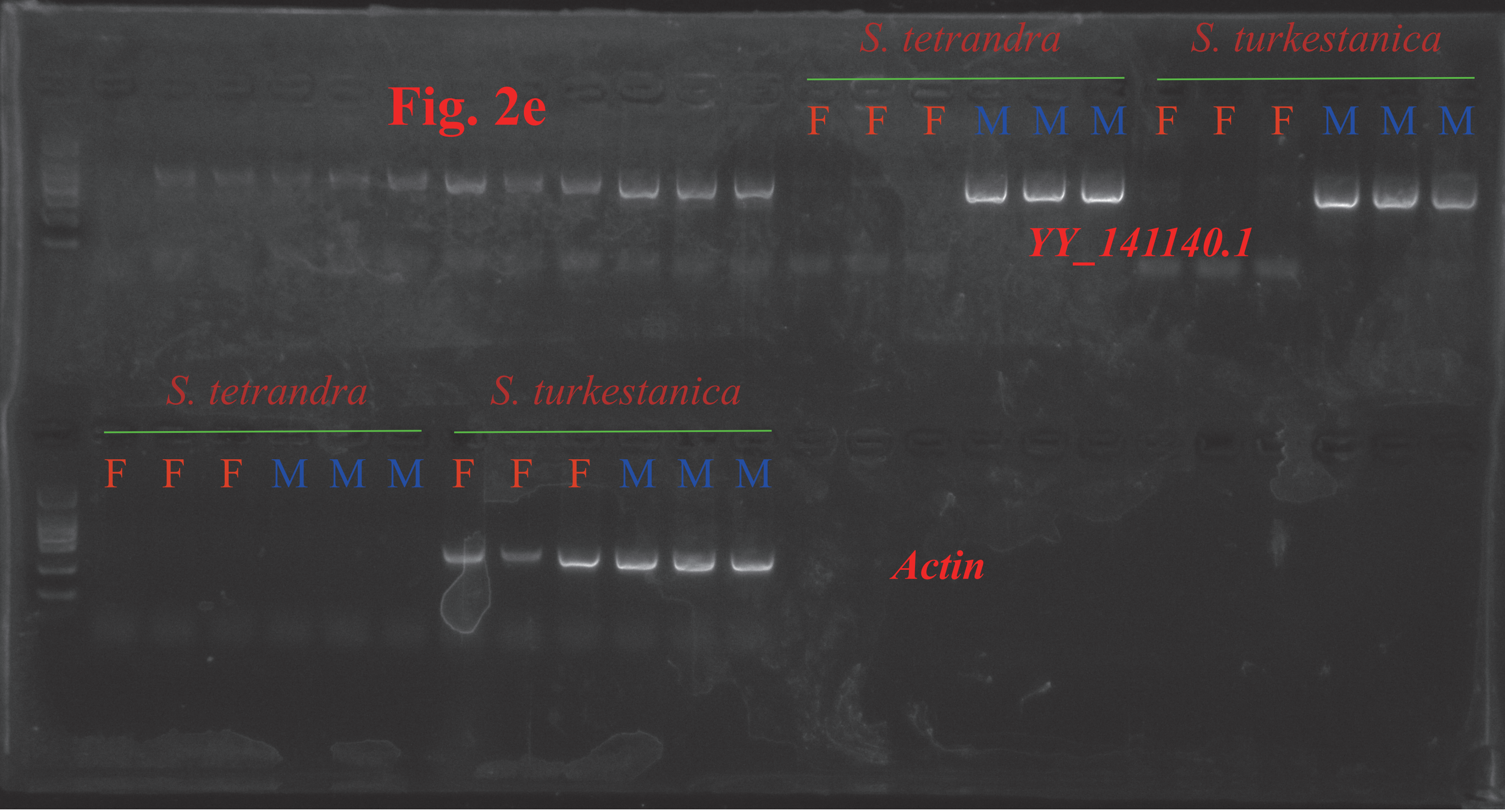
Fig. 2e
S. tetrandra
S. turkestanica
F F F M M M F F F M M M
YY_141140.1
S. tetrandra
S. turkestanica
F F F M M M F F F M M M
Actin
