# Supplementary material for: Remarkable Divergence of the Sex-Linked Region between Two Wild Spinach Progenitors, Spinacia turkestanica and Spinacia tetrandra
Source: Biology (Basel). 2022 Jul 29;11(8):1138. doi: 10.3390/biology11081138 (PMC9404990; doi:10.3390/biology11081138)
Supplement: Supplementary file 1 [file biology-11-01138-s001.zip › Supplementary Figures S1-S7.pdf]

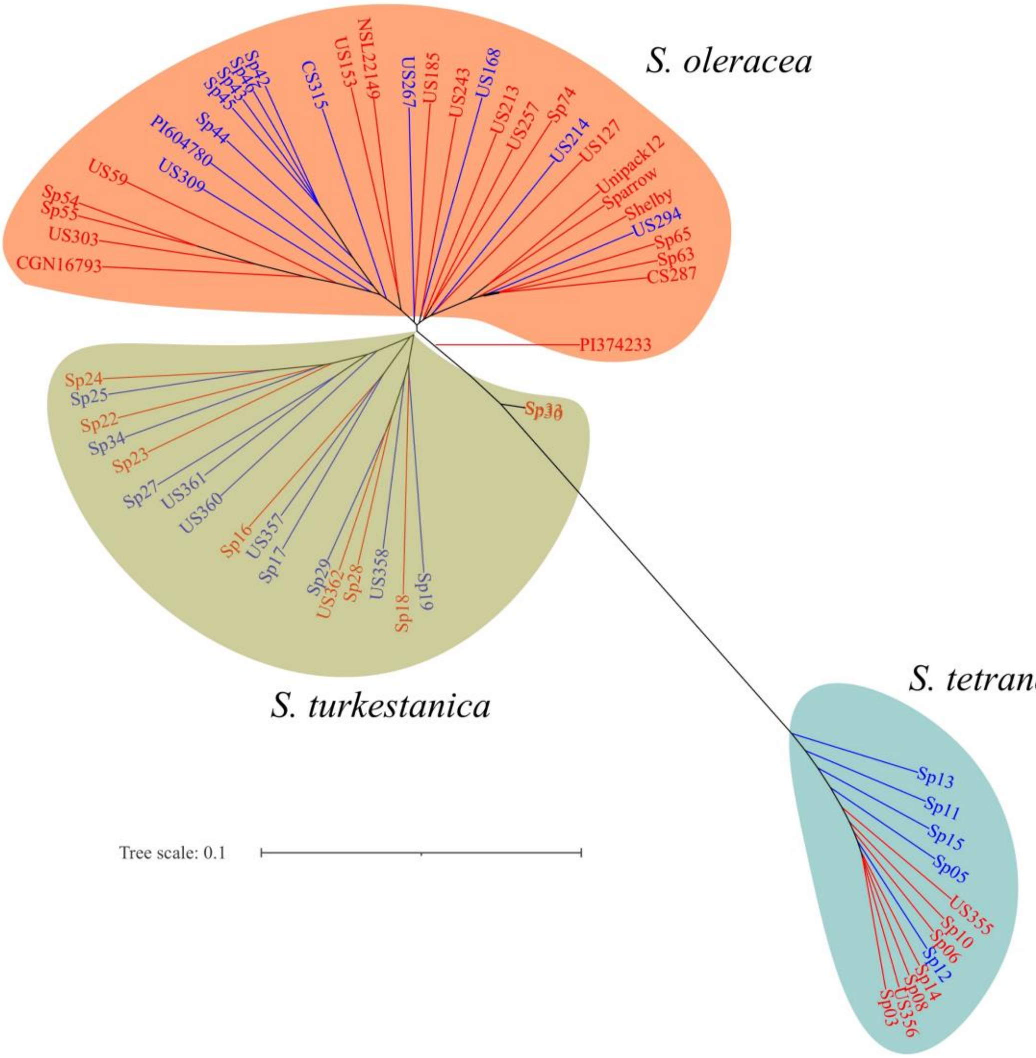

Figure S1. Phylogenetic tree of 63 *Spinacia* accessions based on SNPs within the pseudoautosomal region (PAR).

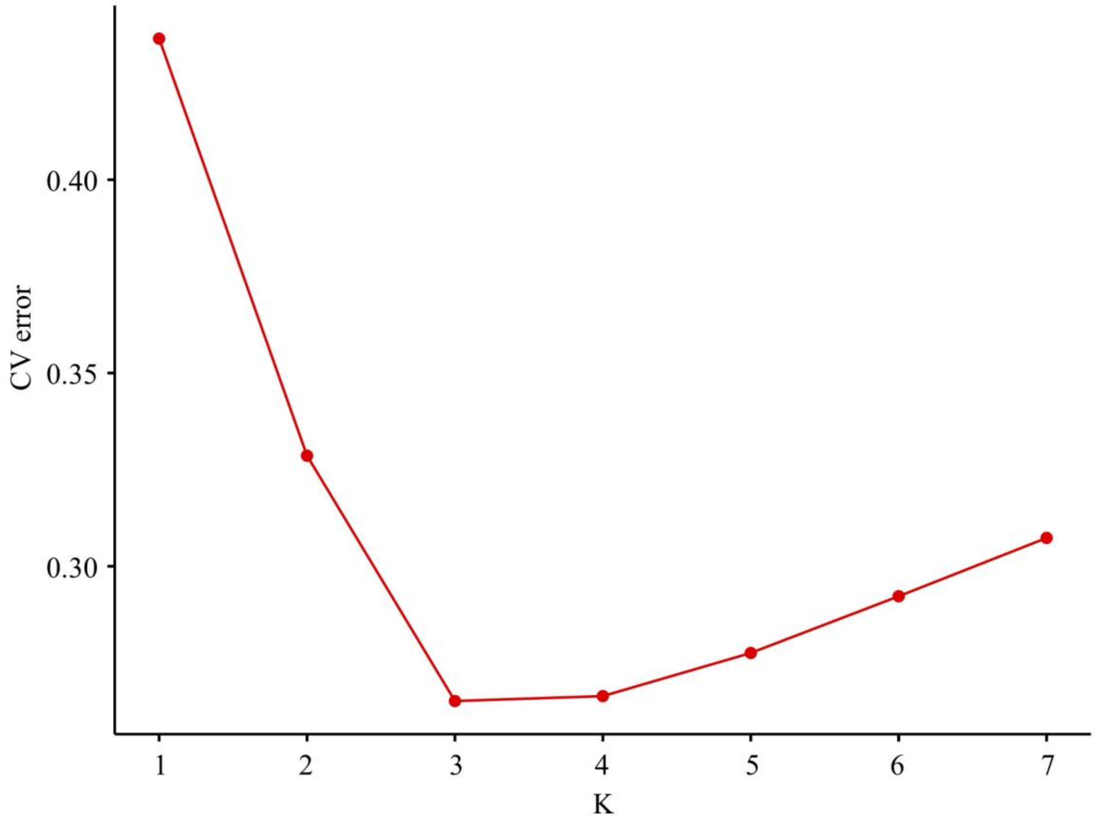

Figure S2. The cross-validation (CV) error for different *K* values in admixture analysis.

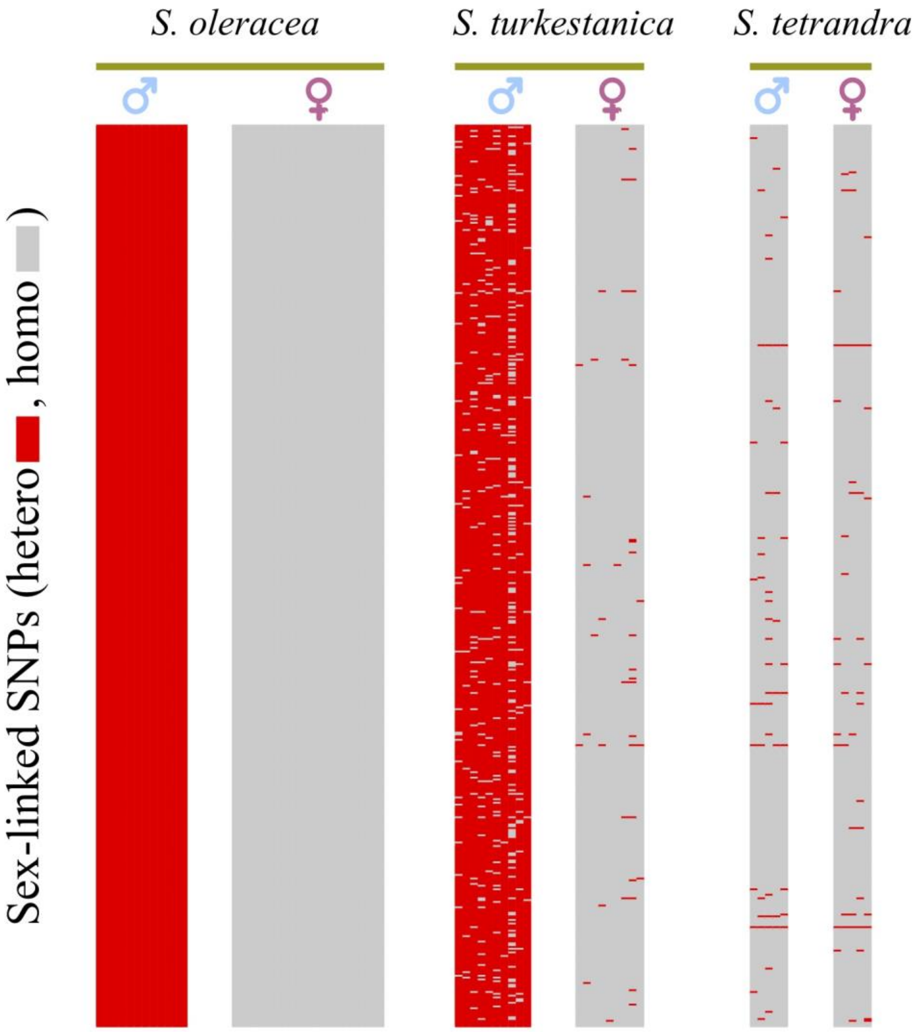

Figure S3. Fully sex-linked SNPs in *S. oleracea*, *S. turkestanica*, and *S. tetrandra.*

Red rectangle indicates heterozygous genotype, while gray rectangle represents homozygous genotype.

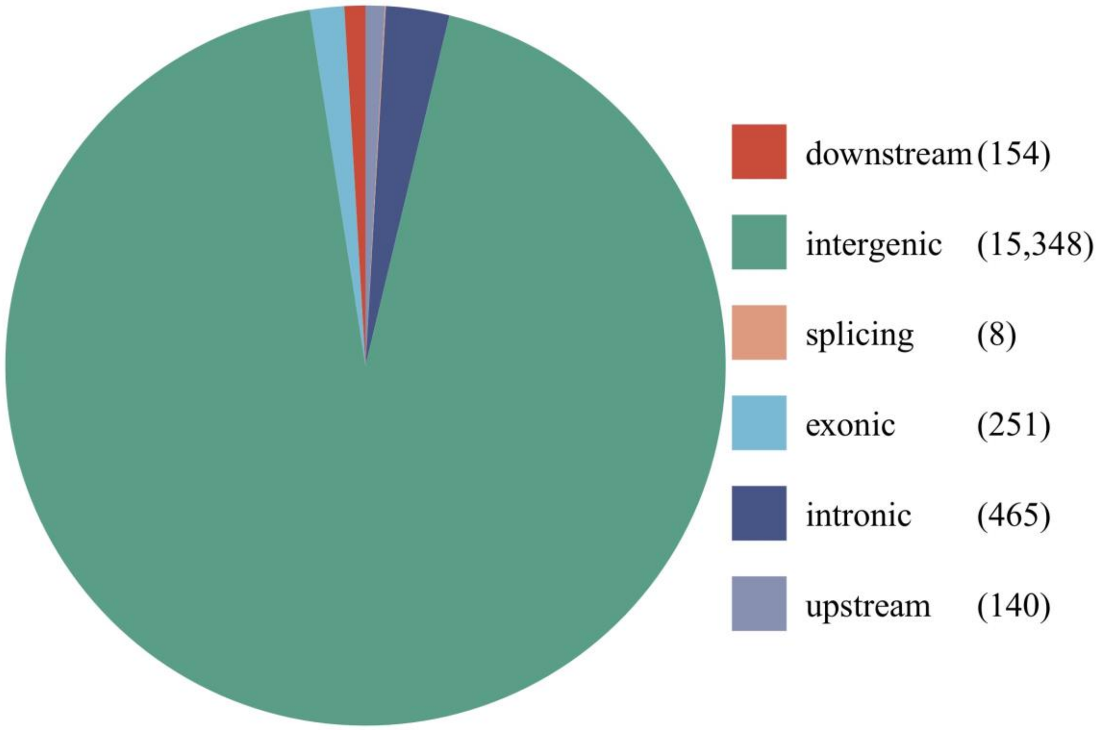

Figure S4. Annotation of SNPs within the Y-duplication region.

Number of SNPs type were shown on the right.

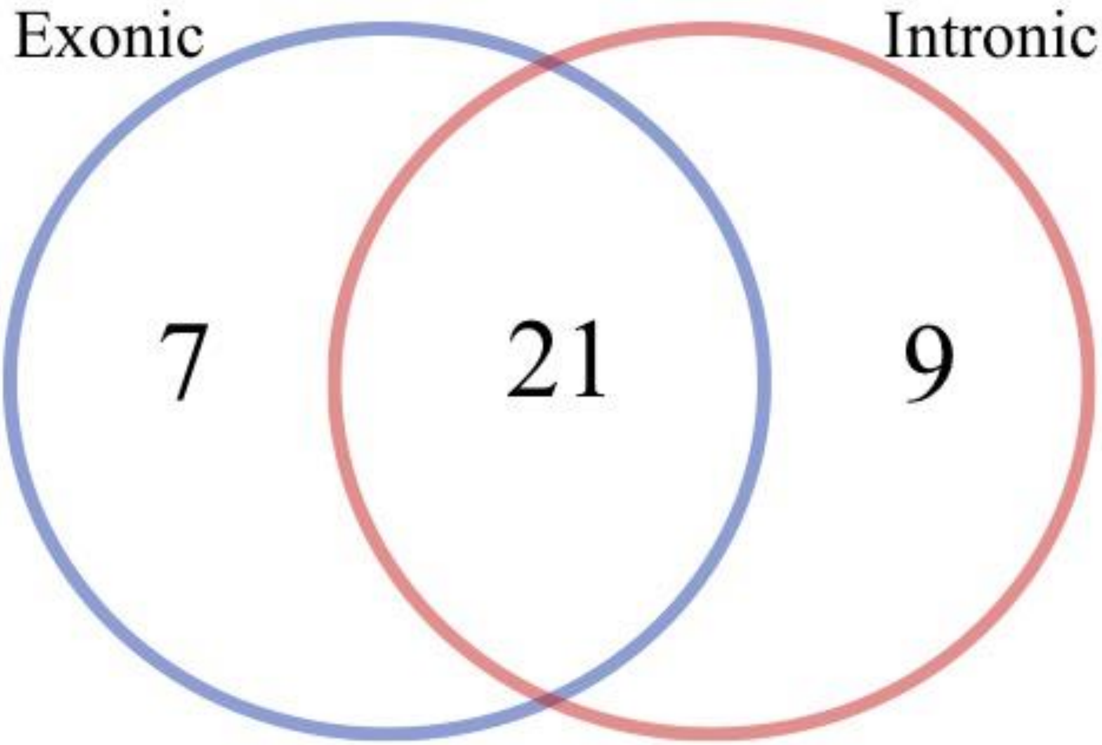

Figure S5. Venn diagram of genes with exonic or intronic SNPs in the Y-duplication region.

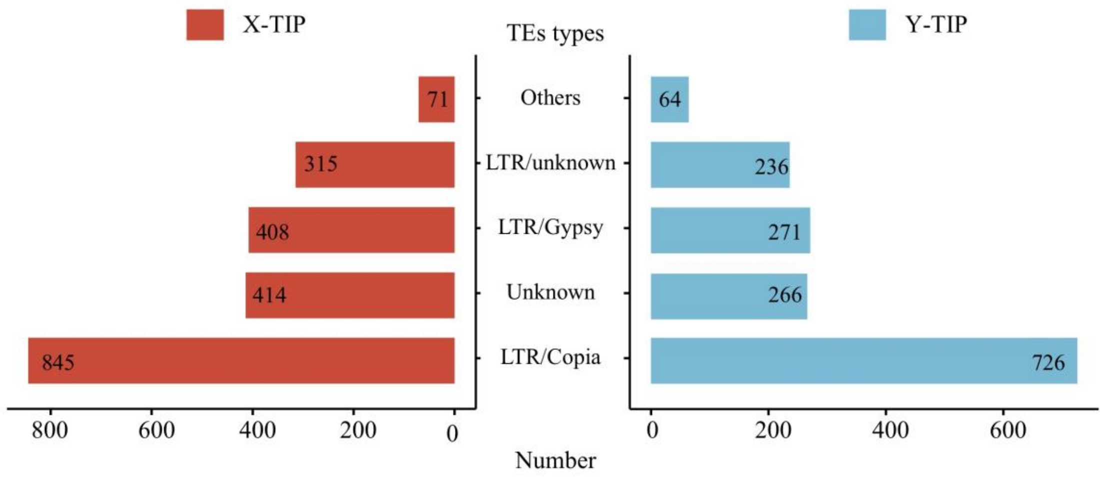

Figure S6. Number of detected TIPs per TE family.

X-TIP represent TIP on the X chromosome. Y-TIP represent TIP on the Y chromosome.

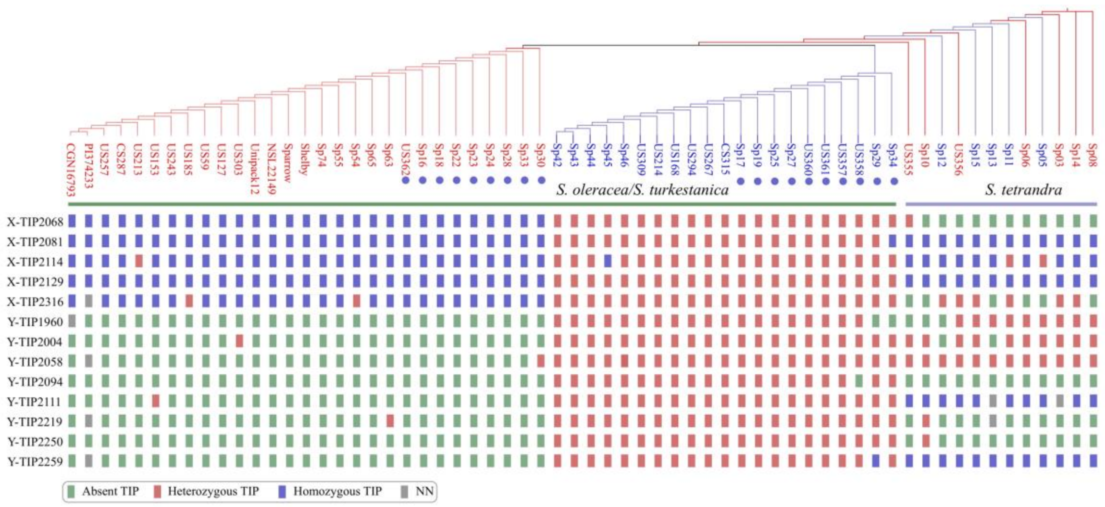

Figure S7. Phylogenetic tree based on 13 conserved TIPs in 62 *Spinacia* accessions.

The accession labelled with red and blue indicate female and male, respectively. The *S. turkestanica* accessions were marked with blue dots. NN: missing.
